# AI‐Powered Virtual Reality in Acne Vulgaris

**DOI:** 10.1111/jocd.70104

**Published:** 2025-03-06

**Authors:** Mohamad Goldust

**Affiliations:** ^1^ Department of Dermatology Yale University School of Medicine New Haven Connecticut USA

**Keywords:** acne vulgaris, artificial intelligence, virtual reality

Dear Editor,

Acne vulgaris is a common skin condition that affects millions of people worldwide and significantly impacts individuals' physical, emotional, and social well‐being. While traditional treatments such as topical therapies, oral medications, and procedural interventions are essential, they often do not fully address all aspects of the condition [1]. However, recent advancements in artificial intelligence (AI) and virtual reality (VR) are expanding acne management by providing innovative tools for diagnosis, treatment, and patient education [2, 3, 4].

## 1 Innovative Applications in Acne Management

AI‐powered applications, such as AcneAI, utilize machine‐learning algorithms and advanced image recognition to accurately detect acne lesions, assess their severity, and predict treatment outcomes. These tools analyze large datasets, enabling precise evaluations and personalized care plans. For example, AcneAI's lesion detection and severity grading provide clinicians with valuable insights that facilitate both diagnosis and treatment (Table 1).

**TABLE 1:** Currently used AI‐powered virtual reality applications in the management of acne vulgaris.

App name	Key features	Functionality	Clinical relevance
AcneAI	AI‐based acne analysis and personalized treatment suggestions integrated into AR/VR environments	Analyzes skin in real‐time, providing instant treatment plans and virtual simulations of outcomes	Improves diagnosis accuracy and speeds up personalized treatment selection
Viveport Human Skin VR	Immersive VR tool for exploring human skin anatomy and pathophysiology, including acne lesions	Enables a detailed 3D visualization of skin layers and conditions like acne	Assists in patient education, training for dermatology professionals, and visualizing the impact of treatments
EgoTouch VR/AR	AI‐integrated AR/VR platform can be used for acne visualization and treatment customization	Provides an interactive experience where patients can “touch” and explore virtual acne treatments	Enhances patient understanding and engagement by simulating the tactile and visual effects of treatments
DermaAI	Advanced skin imaging using AI for acne severity grading	Provides acne assessments and predicts treatment outcomes	Helps dermatologists facilitate the diagnosis and treatment of acne vulgaris

AI‐powered virtual reality (VR) is emerging as an innovative tool in managing acne vulgaris, combining advanced diagnostic capabilities with interactive patient engagement. AI algorithms analyze high‐resolution skin imagery to identify acne subtypes, assess severity, and customize treatment recommendations with substantial precision. When integrated with VR, these technologies offer unique patient experiences such as virtual “before‐and‐after” visualizations of treatment effects that build trust and improve adherence. For instance, VR simulations allow patients to explore 3D models of their skin, demonstrating the impacts of interventions like isotretinoin or laser therapy. This personalized approach not only enhances the clinician‐patient relationship but also enables individuals to make informed decisions about their care.

VR further enhances these capabilities by creating interactive experiences. It simulates treatment effects on detailed 3D skin models, allowing both dermatologists and patients to visualize outcomes before starting therapy. This approach provides patient trust and promotes collaborative decision making.

## 2 Enhancing Patient Education and Engagement

For adolescents and young adults who are the demographics most affected by acne, education is crucial for adhering to treatment plans. AI‐driven visualizations and VR modules effectively address this need. Virtual environments can demonstrate the progression of untreated acne or simulate the effects of therapeutic interventions, helping to set realistic expectations and emphasize the importance of adherence to treatment.

Interactive VR tools can also guide users through skincare routines and lifestyle modifications, such as dietary changes. By making education engaging and relatable, these technologies enhance patient motivation and support long‐term compliance with prescribed regimens.

## 3 Current Challenges

Despite their potential, several barriers inhibit the widespread adoption of AI and VR in acne management:

1. **Cost and accessibility**: Developing and implementing these advanced tools requires significant investment. Limited availability in underserved or remote areas exacerbates disparities in care.
2. **Training needs**: Healthcare providers must receive specialized training to effectively use AI and VR technologies, highlighting the need for structured educational programs.
3. **Data privacy and ethics**: Ensuring robust data protection and the ethical use of sensitive dermatological information is essential.
4. **Validation of effectiveness**: Large‐scale clinical trials are necessary to validate the efficacy and cost‐effectiveness of these technologies in dermatological practice [5].

## 4 Emerging Innovations

Several promising innovations could reshape acne management:

- **Remote consultations**: Virtual dermatology platforms that incorporate AI and VR, such as telehealth solutions for rural areas, can provide personalized treatment plans while improving access to care.
- **Behavioral interventions**: Interactive VR experiences can encourage healthy habits, such as proper skincare routines and dietary changes, complementing medical treatments.
- **Emotional support**: AI and VR programs can address the psychosocial impact of acne. Guided VR sessions offering mindfulness exercises or cognitive‐behavioral therapy can help patients manage stress and anxiety related to the condition.

## 5 Conclusion

AI and VR technologies are redefining acne care by enhancing diagnostic accuracy, engaging patients, and optimizing treatment outcomes. However, addressing challenges related to cost, accessibility, training, and ethical considerations is crucial for their widespread adoption.

## Disclosure

We confirm that the manuscript has been read and approved by all the authors, that the requirements for authorship as stated earlier in this document have been met and that each author believes that the manuscript represents honest work.

## Consent

Informed consent is unnecessary for this work.

## Conflicts of Interest

The author declares no conflicts of interest.
